# Effects of Dominant Fungi on Wheat Quality During Storage

**DOI:** 10.3390/foods15091595

**Published:** 2026-05-05

**Authors:** Xiao He, Jin-Qi Zhao, Bing Wu, Yuan-Yuan Fan, Min Zhang, Qiong Wu, Yu-Rong Zhang, Dong-Dong Zhang, Hai-Jie Li

**Affiliations:** 1 Engineering Research Center of Grain Storage and Security of Ministry of Education, School of Food and Strategic Reserves, Henan University of Technology, Zhengzhou 450001, China; 2 Sinograin Zhengzhou Depot Co., Ltd., Zhengzhou 451150, China; 3 School of Public Health, Hebei Key Laboratory of Occupational Health and Safety for Coal Industry, North China University of Science and Technology, Tangshan 063210, China

**Keywords:** wheat, fungi, wheat storage quality, grain spoilage

## Abstract

To reveal the mechanism underlying the effects of dominant spoilage fungi on wheat quality during storage and provide a theoretical basis for targeted microbial control in wheat storage, this study characterized the structural features of fungal communities on the surface of stored wheat and at different depths of the grain bulk *via* high-throughput sequencing. Additionally, screening was performed for stably existing dominant spoilage fungi in a wheat storage environment. Subsequently, four isolated dominant spoilage fungal strains, *Fusarium lateritium*, *Aspergillus niger*, *Penicillium citrinum* and *Talaromyces islandicus*, were back-inoculated onto wheat kernels sterilized by ^60^Co gamma irradiation. Simulated storage trials were conducted at 28 °C and 80% relative humidity to investigate their impacts on wheat quality. The results show that *F. lateritium* and *A. niger* exhibited faster growth rates and were able to colonize the entire surface of wheat kernels within 8 days. After infection by these two fungi, wheat superoxide dismutase (SOD) activity decreased by 33.83 U/g and 21.90 U/g, peroxidase (POD) activity decreased by 1408 U/(g·min) and 745 U/(g·min), and electrical conductivity (EC) increased by 11.17 μS/(cm·g) and 7.74 μS/(cm·g), respectively. After 10 days of storage, *A. niger* significantly reduced the water absorption of wheat gluten to 175.91% and elevated the fatty acid value to 74.20 mg/100g, rendering the wheat unsuitable for storage. *P. citrinum* exerted the most significant effect on the solvent retention capacity (SRC) of wheat flour in water, sucrose, sodium carbonate, and lactic acid solutions. This study clarified the screening criteria for dominant spoilage fungi in stored wheat, as well as the threshold values and differential characteristics of the impacts of different dominant spoilage fungi on wheat quality, providing critical theoretical support for targeted microbial control during wheat storage.

## 1. Introduction

Wheat is one of the most widely cultivated cereal crops with the largest consuming population worldwide. With an annual planted area of over 220 million hectares and an annual yield of nearly 790 million tons globally, it supplies dietary calories and protein for more than one-third of the world’s population, serving as a staple food crop critical to safeguarding global food security [1]. Among wheat species, durum wheat, the dominant cultivated crop in North Africa and countries along the Mediterranean coast, is the core source of carbohydrates, protein, and minerals in the dietary pattern of local residents [2]. The stability of its production and storage is directly linked to regional food supply security, and numerous recent studies in this region have well documented the fungal infection dynamics and mycotoxin contamination characteristics of local stored wheat, identifying *Aspergillus* and *Fusarium* as the dominant contaminating fungal groups, with the mycotoxin detection rate significantly correlated with storage temperature and relative humidity [3,4].

Wheat undergoes a storage period ranging from months to years between harvest and consumption. During this period, wheat kernels maintain physiological and metabolic activity while being vulnerable to infection by environmental microorganisms. Fungi in storage environments are divided into field residual pathogenic fungi represented by *Fusarium* head blight pathogens and saprophytic spoilage fungi dominated by *Aspergillus*, *Penicillium*, and *Alternaria* [5]. Fungi colonized on stored wheat not only consume key nutrients such as starch and protein, resulting in reduced thousand-kernel weight, germination loss and quality deterioration, but also produce various mycotoxins including deoxynivalenol, aflatoxins, and ochratoxins, posing severe threats to human and animal health [6].

In recent years, global climate change-driven rising temperatures, altered precipitation patterns, and elevated atmospheric CO_2_ concentrations have exerted a profound impact on fungal infection risks in stored wheat [7]. Climate warming increases storage environment temperature, enhancing the fungal reproduction rate and pathogenicity, expanding the suitable growth range of spoilage fungi, and accelerating fungal community succession. Meanwhile, wheat harvested under drought and heat stress exhibits impaired seed coat structure and physiological resistance, further exacerbating the risk of fungal contamination and emerging mycotoxin production during storage [3]. Existing studies confirm that each 1 °C increase in temperature can increase the mycotoxin production rate in stored wheat by 15% to 20%, making climate change a key driver of aggravated fungal contamination [8].

**Figure 1:**
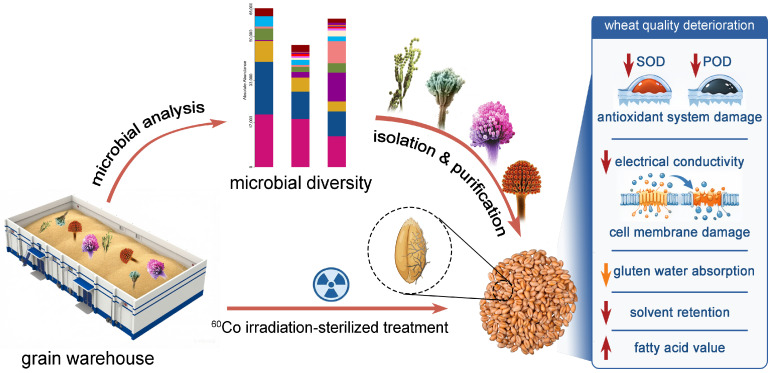
Schematic illustration of sample acquisition and analysis of microbial diversity.

At present, multiple strategies have been applied for stored wheat fungal control, among which biocontrol has received extensive attention for its eco-friendliness and antifungal efficacy [6]. However, the inhibitory efficacy of different antagonistic microorganisms varies widely against different fungal species, and clarifying the quality damage degree of different fungi is an essential prerequisite for targeted control [9]. To date, most studies have focused on fungal co-infection effects and descriptive analysis of fungal community structure, failing to elucidate the causal relationship between individual fungal species and wheat quality deterioration, with the mechanism of quality damage caused by single dominant fungi remaining poorly understood. To address this gap, this study characterized fungal communities in stored wheat at different grain bulk depths via high-throughput sequencing, screened core dominant spoilage fungi, and conducted artificial back-inoculation assays to clarify their deterioration effects on wheat quality, aiming to provide a theoretical basis for targeted fungal control during wheat storage (Figure 1).

## 2. Materials and Methods

### 2.1. Materials and Reagents

Samples for the analysis of depth-dependent microbial community changes were obtained from stored wheat at the National Grain Reserve Depot in Zhengzhou City, Henan Province. DNA molecular weight marker (100–2000 bp), Ezup Column Fungal Genomic DNA Extraction Kit, agarose, ribonuclease A, and 2× PCR Master Mix were purchased from Sangon Biotech (Shanghai) Co., Ltd. (Shanghai, China). Potato Dextrose Agar (PDA) was purchased from Beijing Aoboxing Bio-Tech Co., Ltd. (Beijing, China). FastDNA^®^ Soil DNA Extraction and Purification Kit was purchased from Shanghai Anbei Medical Device Trading Co., Ltd. (Shanghai, China). Trichloroacetic acid (TCA) was purchased from Luoyang Chemical Reagent Factory (Luoyang, China). Superoxide Dismutase (SOD) Activity Assay Kit was purchased from Beijing Solarbio Science & Technology Co., Ltd. (Beijing, China). All reagents used in this study were at least of analytical grade, and double-distilled water (ddH_2_O) was used for all experimental procedures.

### 2.2. Analysis of Microbial Diversity on Wheat Kernel Surfaces

High-throughput sequencing was performed to characterize the baseline structural features of fungal communities on the surface of stored wheat kernels, clarify the composition of core fungal taxa in the wheat storage environment, and define the species pool that forms the fundamental basis for the subsequent targeted screening of dominant spoilage fungi.

Sample pretreatment was performed with minor modification to the method established by Xu et al. [10]. Briefly, 50 g wheat samples were immersed in sterile water, shaken at 180 rpm for 30 min, filtered through three layers of sterile gauze, and centrifuged at 10,000× *g* for 10 min at 4 °C. The supernatant was discarded, and the resulting microbial pellets were stored at −80 °C until subsequent DNA extraction. All utensils used in the experiment were pre-autoclaved at 121 °C for 20 min prior to use.

DNA Extraction and High-Throughput Sequencing. Total genomic DNA was extracted using the FastDNA^®^ Soil Kit per the manufacturer’s instructions, followed by quality verification and spiking with gradient-copy controls. The fungal ITS1 rRNA gene region was amplified for high-throughput sequencing on an Illumina NovaSeq 6000 platform.

Sequencing Data Analysis. Raw Illumina sequencing reads were processed in QIIME2 for trimming, quality control, amplicon sequence variant (ASV) calling, and UNITE-based taxonomic annotation, with spike-in controls used for absolute abundance calibration. α-diversity, one-way analysis of variance (ANOVA), and data visualization were performed using R (version 3.5.1) with the vegan (v2.5.6) and ggplot2 (v3.3.0) packages [11,12].

### 2.3. Depth-Dependent Microbial Changes in Stored Wheat in the Warehouse

Wheat samples collected from different depths within the grain bulk were used to characterize differences in microbial community structure, clarify the enrichment characteristics of fungi in high-risk mildew-prone areas during grain storage, and perform systematic inter-group analyses of diversity, community structure, species distribution, and functional guilds. This work provides a solid ecological basis for the subsequent targeted screening of dominant spoilage fungi. All experimental procedures, including sample pretreatment, total genomic DNA extraction, ITS and 16S rRNA gene amplification, high-throughput sequencing, and raw data processing, were identical to those described in the standardized methods detailed in Section 2.2. Additional in-depth analyses were conducted, including a one-way ANOVA for α-diversity difference testing, construction of sample clustering trees, Principal Coordinates Analysis (PCoA) based on the Jaccard distance matrix, ASV distribution analysis, and prediction of fungal functional guilds using the FUNGuild database. Briefly, the annotated fungal taxa were matched with the guild classification database, and the trophic modes and specific functional guilds of each fungal taxon were classified [10].

### 2.4. Isolation and Identification of Dominant Fungi

Based on the fungal community diversity results from the aforementioned microbial survey of wheat kernel surfaces and different grain bulk depths, dominant spoilage fungi were screened from wheat samples according to four pre-established core criteria: (1) stable detection across all community survey samples; (2) well-documented cereal spoilage potential; (3) assignment to pathotrophic or saprotrophic functional guilds; and (4) high isolation frequency and colony abundance in culture-dependent assays. Target strains were obtained via gradient dilution and plating on PDA medium, followed by repeated single-colony isolation and purification. Taxonomic identification was conducted via amplification and Sanger sequencing of the ITS rRNA gene, with species identity confirmed through BLAST alignment against the NCBI nucleotide database (MEGA 11). Validated pure strains were cryopreserved in 30% (*v*/*v*) aqueous glycerol solution at −80 °C for subsequent artificial inoculation assays on wheat [13].

### 2.5. Infection of Stored Wheat by the Isolated Dominant Fungi

To clarify the independent infection effect and quality deterioration capacity of the obtained dominant spoilage fungi on stored wheat, target glycerol-preserved pure strains were first activated on PDA medium and incubated at 28 °C until abundant spores were produced. Purified spore suspensions were prepared by rinsing the medium surface with sterile water, filtering to remove residual mycelia, counting concentration via hemocytometer, and adjusting to a standardized 1 × 10^7^ CFU/mL. ^60^Co irradiation-sterilized (12 kGy) wheat samples were inoculated with the spore suspension under aseptic conditions, with sterile water as the blank control. All samples were stored at 28 °C and 80% relative humidity, with serial sampling performed on days 2, 4, 6, 8 and 10 after inoculation, respectively [14]. After 2 days of pre-culture under the above simulated storage conditions, the sterility of the irradiation treatment and the success of fungal inoculation were verified via total fungal colony count assay (described in Section 2.6). No fungal colonies were detected in the ^60^Co irradiation-sterilized non-inoculated control, while the total fungal colony counts of all inoculated groups ranged from 5.6 × 10^3^ CFU/g to 7.0 × 10^3^ CFU/g. These results confirm that the irradiation treatment achieved complete sterilization, and fungal inoculation was successful in all experimental groups.

### 2.6. Total Fungal Colony Count

The abundance of culturable fungi on wheat kernels was quantified via colony-forming unit (CFU) counting. The detailed procedure was as follows: (1) a 25 g wheat sample was fully homogenized with 225 mL sterile physiological saline (standard diluent specified in the standard) at 8000 r/min–10,000 r/min for 1 min–2 min to prepare a 1:10 sample homogenate, followed by four successive 10-fold serial dilutions; (2) 100 μL of each appropriate dilution was spread evenly on PDA plates in triplicate, with blank diluent set as negative control; (3) plates were incubated upside down at 28 °C for 5 days; and (4) plates with 30 CFU–300 CFU colonies were selected for counting, plates with <30 CFU were recorded with exact values, and those with >300 CFU were marked as too numerous to count. The total fungal count per gram of wheat was calculated using the standard formula, and the test was deemed invalid if colonies were detected in the blank control. Meanwhile, wheat chromaticity was measured using a CM-5 colorimeter, with three replicates per group to minimize random errors, and the average value was used for subsequent data analysis.

### 2.7. Physiological and Biochemical Properties Determination of Wheat

Chromaticity analysis of wheat samples was performed using a CM-5 colorimeter, with minor modifications to the method reported by Wang et al. [15].

MDA content was determined with minor modifications to the method described by Tian et al. [16]. Briefly, approximately 1.00 g of whole wheat flour was mixed with 5.00 mL of 10% (*w*/*v*) TCA, then centrifuged at 10,000× *g* for 10 min at 4 °C. Subsequently, 2.00 mL of the supernatant was mixed with 2.00 mL of 0.6% (*w*/*v*) thiobarbituric acid (TBA). The mixture was boiled for 15 min, rapidly cooled to room temperature (25 °C), and centrifuged at 10,000× *g* for 10 min at 4 °C. The absorbance of the supernatant was measured at 450 nm, 532 nm, and 600 nm, with TCA solution used as the blank control. MDA content was expressed as mmol/kg fresh weight.

Wheat kernel membrane permeability was assessed by measuring electrical conductivity [17], with minor modifications to the method of Liu et al. [18]. Briefly, 25 wheat kernels of uniform size were weighed before the experiment (to ensure that the difference in weight between groups was less than 5%), washed three times with deionized water, and immersed in 50 mL of deionized water for 12 h. Finally, the conductivity of the wheat samples was measured using a conductivity meter.

POD activity of wheat was determined with minor modifications to the method reported by Ahmad et al. [19]. Briefly, 1.00 g of whole wheat flour and 10.00 mL of phosphate buffer (0.05 mol/L, pH 6.0) were combined and centrifuged at 10,000× *g* for 10 min at 4 °C. Next, 1.00 mL of supernatant was added to a reaction mixture containing 2.00 mL of phosphate buffer, 1.00 mL of H_2_O_2_ solution (2%, *v*/*v*), and 1.00 mL of guaiacol solution (2%, *v*/*v*). The absorbance of the mixture was measured at 470 nm every minute for 5 min. One unit of POD activity was defined as a 0.01 change in absorbance at 470 nm per minute, and POD activity was expressed as U/kg fresh weight.

### 2.8. Quality Determination of Fungi-Infected Wheat and Statistical Analysis

Key storage and processing quality indicators of wheat kernels infected with different fungi were determined with minor modifications to standard and previously reported methods.

Wet gluten content and gluten water absorption were measured according to the method described by Ravinder et al. [20]. Briefly, 10.00 g of whole wheat flour was mixed with 5 mL of 20 g/L NaCl solution to form a dough. The dough was washed with 20 g/L NaCl solution at a flow rate of 50 mL/min for 8 min, followed by rinsing with distilled water until the eluate showed no blue color in KI-I_2_ titration. The remaining viscoelastic gluten mass was drained of excess surface water to obtain wet gluten, which was then dried in a dedicated gluten dryer for 300 s to obtain dry gluten. Wet gluten content was expressed as a percentage of the initial flour mass, and gluten water absorption was calculated as the ratio of the mass difference between wet and dry gluten to the dry gluten mass.

Fatty acid value was determined via titration with minor modifications to the method of Tian et al. [16]. Briefly, 10.00 g of whole wheat flour was mixed with 50 mL of anhydrous ethanol in a stoppered conical flask, shaken for 30 min, and filtered. Then, 25 mL of the filtrate was mixed with 25 mL of anhydrous ethanol and 25 mL of thymol blue indicator, and the mixture was titrated with 0.01 mol/L KOH standard solution until the lower ethanol layer remained yellowish green for 30 s. The titration volumes of the sample and blank control were recorded, and the fatty acid value was calculated and expressed as mg KOH per 100 g of dry sample (mg/100 g).

Solvent retention capacity (SRC) was measured according to the method of Magallanes Kumar et al. [21]. Four solvents were used for the assay: 5% (*w*/*v*) sodium carbonate solution, 50% (*w*/*v*) sucrose solution, 5% (*w*/*v*) lactic acid solution, and distilled water. Briefly, 5.000 g of wheat flour was weighed and placed into a 50 mL centrifuge tube, and 25.00 g of the corresponding solvent was added. The tube was shaken vigorously until the mixture was homogeneous, and it was left to stand for 20 min for hydration (with 5 s rapid shaking at 5, 10, 15 and 20 min). The tube was centrifuged at 1000× *g* for 15 min immediately after the final shaking step. The supernatant was discarded, and the tube was inverted on filter paper for 10 min to drain residual liquid. The SRC value of wheat flour for each solvent was calculated based on the total mass of the centrifuge tube, lid and residual flour gel.

All indicators of wheat samples were analyzed using a one-way ANOVA. Duncan’s multiple range test was used to compare significant differences between means at a significance level of *p* ≤ 0.05. All statistical analyses were performed using SPSS 26.0 software, and figures were created using Origin 2018 software. All experiments were conducted in triplicate, and the mean values were used for data analysis and plotting.

### 2.9. Statistical Analysis

The data employed for plotting within this study were the mean values resulting from three biological replicates and three technical replicates of all experiments. The error bars were determined by calculating the standard deviation derived from the replicate experimental data. Basic data visualization graphs were produced using Origin 2018 software. Bioinformatic analysis of fungal high-throughput sequencing data, as well as α-diversity analysis and related visualization, were performed using QIIME2 and R (version 3.5.1) software, with the vegan (v2.5.6) and ggplot2 (v3.3.0) packages applied for community diversity analysis and plotting. Statistical significance analysis was executed utilizing SPSS 26.0 software. One-way ANOVA followed by Duncan’s multiple range test was used for the comparison of significant differences between groups, with significance levels set at *p* ≤ 0.05 (indicating significant differences) and *p* ≤ 0.01 (indicating highly significant differences).

## 3. Results and Discussion

### 3.1. Analysis of Fungal α-Diversity

**Figure 2:**
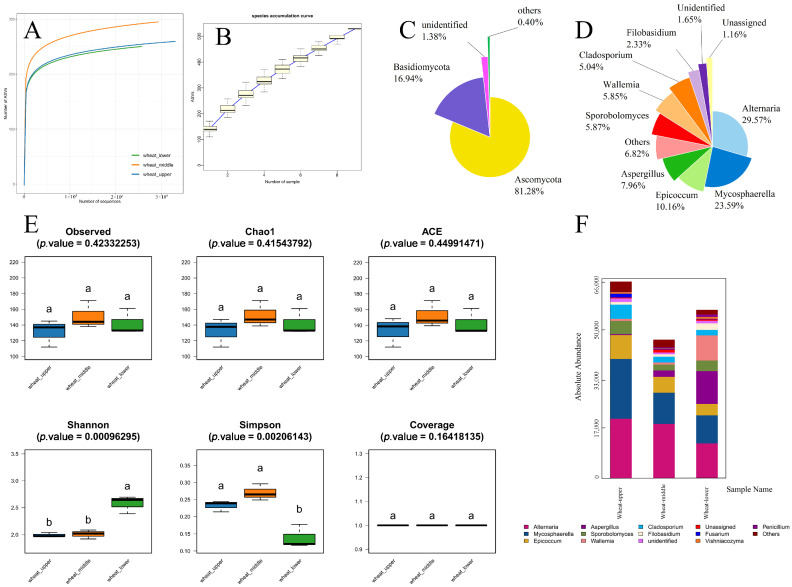
Comprehensive characterization of fungal communities in stored wheat across different grain bulk depths. (**A**) Rarefaction curves of fungal ASVs. (**B**) Species accumulation curve. (**C**) Relative abundance of fungal communities at the phylum level. (**D**) Relative abundance of dominant fungal communities at the genus level. (**E**) α-diversity indices of fungal communities across different grain bulk depths. (**F**) Absolute abundance of dominant fungal genera in wheat from different grain bulk depths. Note: The same letters in the figure indicate no significant difference between the two groups.

Rarefaction curves of fungal communities in wheat samples reached a plateau with increasing sequencing depth (Figure 2A), and species accumulation curves showed a gentle rising trend with no abrupt increase (Figure 2B). The sequencing coverage of all wheat samples was close to 1. For α-diversity indices of the total fungal communities, the observed species index was 141.78, with Chao1 and ACE indices both at 142; the Shannon index was 2.543; and the Simpson index was 0.16 [22].

At the phylum level, *Ascomycota* and *Basidiomycota* were the dominant fungal phyla in wheat samples, with relative abundances of 81.28% and 16.94%, respectively; unidentified and other rare phyla accounted for only 1.78% in total (Figure 2C). At the genus level, *Alternaria* (29.57%) and *Mycosphaerella* (23.59%) were the most dominant genera, followed by *Epicoccum* (10.16%) and *Aspergillus* (7.96%). Low-abundance genera including *Sporobolomyces*, *Wallemia*, *Cladosporium* and *Filobasidium* were also detected (Figure 2D).

The high sequencing coverage and plateaued rarefaction curves confirm that the sequencing depth is sufficient to capture the majority of fungal diversity in stored wheat samples. The absolute dominance of *Ascomycota* is consistent with the ecological characteristics of grain storage environments, where *Ascomycetes* exhibit stronger adaptability to low-moisture and oligotrophic conditions compared to Basidiomycetes. The high relative abundance of *Alternaria* and *Mycosphaerella* reflects the residual effect of field fungal infection, as these two genera are typical field pathogens that can survive on wheat kernels during the early storage period.

The sequencing data obtained in this study are reliable, and the dominant phylum *Ascomycota* is highly consistent with the findings of Solanki et al. [4] on global stored wheat microbiota, which further validates the universality of *Ascomycota* as the core fungal phylum in stored grain ecosystems. Unlike previous studies that focused on fungal community composition [5,22], this study further characterized the vertical distribution patterns of dominant fungal genera at different grain bulk depths, and the core dominant taxa of stored wheat fungi across the entire grain bulk were clarified. Mixed samples from a single depth or random sampling points were analyzed in most previous studies, by which the spatial heterogeneity of fungal communities in actual grain depots cannot be reflected. A more comprehensive understanding of the fungal community structure in stored wheat is provided by this spatial dimension of data, and a solid foundation for subsequent targeted screening of dominant spoilage fungi is laid.

### 3.2. Fungal Diversity of Wheat Across Different Grain Bulk Depths

One-way ANOVA with Bonferroni correction was performed to assess significant differences in fungal α-diversity indices among wheat samples from upper, middle and lower layers of the grain bulk depths, with *p* < 0.05 set as the significance threshold. The sequencing coverage of all samples was close to 1, with no significant difference among the three groups (*p* = 0.164).

For community richness indices (observed species, Chao1, ACE), the highest values were all recorded in the middle layer wheat group, with no statistically significant differences detected among the three groups (all *p* > 0.05). For community diversity and evenness indices, significant differences were observed among groups for both the Shannon index (*p* = 0.001) and Simpson index (*p* = 0.002). The post hoc Bonferroni test revealed that the lower-layer wheat group had a significantly higher Shannon index and lower Simpson index than the upper and middle-layer groups (Figure 2E).

This study systematically clarifies that the middle layer of grain bulk has the highest fungal richness, while the lower layer has significantly higher community diversity. The middle layer is located at the interface between the upper and lower layers, where temperature and humidity conditions are relatively moderate, allowing the coexistence of both aerobic and facultative anaerobic fungi, thus resulting in the highest species richness. The high-humidity microenvironment in the lower layer supports the growth of various fungi, which is the core reason for its higher diversity. This finding indicates that the lower layer of grain bulk should be the key monitoring area for early warning of grain mildew in actual storage operations.

Temporal changes during storage have been mainly focused on in previous studies on fungal diversity in stored wheat, with limited attention paid to spatial differences within the grain bulk [23,24]. It was reported by Li et al. [23] that fungal diversity increased with storage time in laboratory simulation experiments, but the vertical distribution pattern was not investigated. Unlike these studies, it was demonstrated for the first time that significant differences in fungal diversity exist across different grain bulk depths in actual grain depots. The finding that the lower layer has the highest community diversity contradicts the traditional view that the upper layer is the most prone to mildew due to direct contact with air. This discrepancy can be attributed to the fact that most previous studies were conducted under controlled laboratory conditions with uniform temperature and humidity, by which the complex microenvironmental gradients present in actual large-scale grain depots cannot be replicated. A more realistic basis for the layout of monitoring points in grain storage facilities is provided by these results.

### 3.3. Effects of Grain Bulk Depth on Wheat Fungal Community Structure

Fungal genera with relative abundance accounting for more than 1% in the total community were screened for community structure analysis, and the distribution of fungal communities in wheat from different grain bulk depths was visualized via stacked bar chart (Figure 2F). The total absolute abundance of fungal communities in wheat samples ranked in descending order as follows: wheat-upper (WU) > wheat-lower (WL) > wheat-middle (WM).

*Alternaria* was the dominant genus in all three sample groups, with relative abundances of 30.32%, 39.25% and 20.74% in WU, WM and WL, respectively. *Mycosphaerella* was the second core dominant genus in the WU and WM groups, with relative abundances of 30.32% and 22.47%, respectively, while *Aspergillus* accounted for 19.44% of the total fungal community in the WL group, far higher than that in the other two groups. For the WU group, the high-abundance dominant genera also included *Epicoccum* (12.12%), *Cladosporium* (7.22%) and *Sporobolomyces* (6.64%). For the WM group, *Epicoccum* (11.51%) was another major dominant genus, followed by *Aspergillus* (4.51%), Sporobolomyces (4.28%) and *Cladosporium* (4.16%), all with relative abundances below 5%. For the WL group, *Wallemia*, *Epicoccum* and *Sporobolomyces* were also the main high-abundance genera in addition to the core dominant taxa mentioned above.

The fungal species composition of wheat in different grain bulk depths is similar, but the relative abundance of dominant genera is significantly different, which is consistent with the laboratory simulation results of Li et al. [23]. This study verified this rule based on actual grain depot samples and found that *Aspergillus* is significantly enriched in the lower layer, which directly explains why the lower layer is more prone to mildew.

The similarity in species composition across different grain depths is consistent with the laboratory simulation results of Li et al. [23], who found that the core fungal species remained stable during storage while their relative abundances changed. However, the significant enrichment of *Aspergillus* in the lower layer was not observed in previous studies, mainly because their experimental systems did not reproduce the vertical moisture gradient that exists in actual grain depots. Unlike previous laboratory-based studies, this ecological rule was verified based on samples collected from a national grain reserve depot. The finding that *Aspergillus* is significantly enriched in the lower layer provides a direct ecological explanation for the higher mildew risk in the lower grain layer. Important practical implications for grain storage management are presented by this result, suggesting that targeted monitoring and control measures should be implemented in the lower layer to prevent *Aspergillus* contamination.

### 3.4. Differences in Fungal Community Structure on Wheat Kernels Across Different Grain Bulk Depths

**Figure 3:**
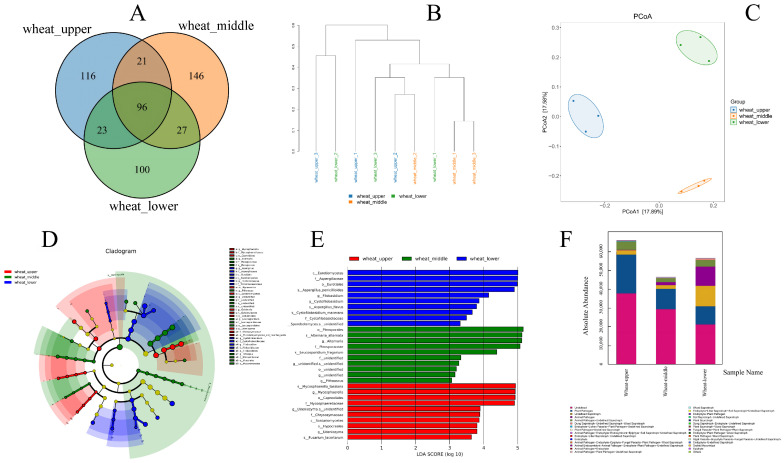
Venn diagram of shared and unique fungal ASVs among groups (**A**). Hierarchical clustering tree based on Bray–Curtis dissimilarity (**B**). PCoA ordination plot based on Jaccard index (**C**). Cladogram of differential fungal taxa from LEfSe analysis (**D**). LDA score distribution of differential fungal biomarkers (**E**). Absolute abundance of functional guilds of fungal communities predicted via FUNGuild (**F**).

Venn diagram analysis showed that a total of 96 core ASVs were shared among the WU, WM and WL groups (Figure 3A). The high-abundance shared ASVs were taxonomically annotated to *Alternaria alternata*, *Epicoccum nigrum* and *Cladosporium tenuissimum*. The number of unique ASVs in the WU, WM and WL groups was 116, 146 and 100, respectively.

Hierarchical clustering analysis based on the Bray–Curtis dissimilarity matrix with the average-linkage method showed that the three biological replicates of each group were preferentially clustered together, and samples from the same grain bulk depth formed an independent cluster (Figure 3B).

PCoA based on the Jaccard index showed that the first two principal coordinates explained 17.89% and 17.58% of the total variation in the fungal community, respectively (Figure 3C). Samples from the WU, WM and WL groups were completely separated on the coordinate axis, with no overlap of confidence ellipses among groups.

Permutational Multivariate Analysis of Variance (PERMANOVA) based on the Jaccard distance matrix confirmed that the difference in fungal community structure among the three groups was statistically significant (*p* < 0.05).

The 96 core shared ASVs among the three groups constitute the core fungal community of stored wheat in this depot, which represents the stable fungal taxa that can survive across different microenvironments within the grain bulk. The presence of unique ASVs in each layer confirms that grain bulk depth has a significant effect on fungal community composition, as different microenvironmental conditions select for specific fungal taxa. The clear separation of samples from different depths in the PCoA plot and hierarchical clustering tree further verifies that the fungal community structure has significant spatial differentiation in different grain depths. This spatial differentiation provides a solid ecological basis for the subsequent screening of dominant spoilage fungi, as it indicates that spoilage fungi may be unevenly distributed within the grain bulk.

Core fungal communities in stored wheat from different geographical regions have been identified in previous studies [4,5], but the core community within a single grain bulk has been rarely investigated. It was reported by Solanki et al. [4] that *Alternaria*, *Aspergillus* and *Penicillium* were the core genera in global stored wheat microbiota, which is consistent with the finding that *Alternaria alternata* is the most abundant core ASV. Unlike these global-scale studies, the spatial variation in fungal communities within a single grain bulk was focused on in this work. The results show that although the core fungal taxa are shared across different depths, each depth has its own unique fungal taxa. This finding challenges the traditional view that the fungal community is homogeneous within a grain bulk and highlights the importance of spatial sampling in stored grain microbial research. Mixed samples from multiple depths were used in most previous studies, by which the spatial heterogeneity of fungal communities may have been masked and an underestimation of the actual diversity may have been caused.

### 3.5. LEfSe Differential Biomarker Analysis of Fungal Communities

Linear discriminant analysis effect size (LEfSe) was performed to identify the characteristic fungal taxa with significant differences among wheat samples from different grain bulk depths (Figure 3D,E). The characteristic fungal taxa in the WU group were mainly represented by the genera *Mycosphaerella*, *Fusarium* and *Capnodiales*; the characteristic taxa in the WM group were dominated by the genera *Alternaria*, *Pleosporaceae* and *Leucosporidium*; and the WL group had significant enrichment of the genus *Aspergillus*, family *Aspergillaceae* and order *Eurotiales* as the core differential biomarkers, with LDA scores (log10) above 4.5.

*Alternaria* was identified as the core biomarker of the middle grain layer and *Aspergillus* as the biomarker of the lower layer by LEfSe analysis, which is consistent with the previous community structure results. The enrichment of field fungi such as *Mycosphaerella* and *Fusarium* in the upper layer reflects the fact that the upper layer is more exposed to external environmental influences and retains more field-derived fungi. The dominance of *Alternaria* in the middle layer is related to its strong adaptability to moderate temperature and humidity conditions. The significant enrichment of *Aspergillus* in the lower layer further confirms that this area is a high-risk zone for storage fungal contamination.

LEfSe analysis has been mainly used in previous studies to identify differential fungal taxa between different storage times or different wheat varieties [25]. For example, *Aspergillus* and *Penicillium* were identified as biomarkers for late storage stages by Li et al. [23], indicating the succession from field fungi to storage fungi over time. Unlike previous studies focusing on the temporal succession of storage fungi, it was found that the succession from field fungi to storage fungi also exists in the vertical space of grain bulk. The upper layer is dominated by field fungi (*Mycosphaerella*, *Fusarium*), while the lower layer is dominated by storage fungi (*Aspergillus*). This spatial succession pattern is analogous to the temporal succession pattern observed in previous studies, which improves the cognition of fungal succession during grain storage. This finding suggests that the grain bulk can be regarded as a “time capsule” where different stages of fungal succession coexist in different spatial locations.

### 3.6. Functional Prediction of Fungal Communities Using FUNGuild

The trophic modes and functional characteristics of fungal communities in wheat from different grain bulk depths were predicted using the FUNGuild database (Figure 3F). A total of seven trophic modes were annotated in the wheat fungal communities, among which Pathotroph, Saprotroph, and Pathotroph–Saprotroph were the core trophic modes for fungi with verified wheat spoilage potential assigned to spoilage-related functional guilds. The absolute abundance of each functional guild varied with grain bulk depth. The total absolute abundance of fungal functional guilds ranked in descending order as WU > WL > WM, which was consistent with the variation trend of total fungal abundance at the genus level.

Undefined trophic mode, Plant Pathogen, Undefined Saprotroph and Animal Pathogen were the dominant functional guilds across all samples. The WU group had the highest absolute abundance of Plant Pathogen among the three groups, while the absolute abundances of Animal Pathogen and Undefined Saprotroph in the WL group were notably higher than those in the WU and WM groups. Meanwhile, the total abundance of saprotrophic fungi in the WU group was higher than that in the middle and lower layers [24].

It was shown by FUNGuild prediction that Pathotroph and Saprotroph are the core trophic modes of stored wheat fungi, which is consistent with the functional characteristics of grain spoilage fungi. Pathotrophic fungi mainly cause plant diseases and reduce grain vitality, while saprotrophic fungi decompose organic matter in grain and cause quality deterioration. The higher abundance of Plant Pathogen in the upper layer is consistent with the enrichment of field pathogenic fungi in this layer. The significantly higher abundance of saprotrophic functional guilds in the lower grain layer confirms from the functional perspective that the lower layer is a high-risk area for wheat storage deterioration, as saprotrophic fungi are the main drivers of grain spoilage.

The application of FUNGuild for functional prediction of stored grain fungal communities has become increasingly common in recent years [25]. It was reported by Tripathi et al. [24] that Pathotroph and Saprotroph were the dominant trophic modes in agricultural ecosystems, which is consistent with the results obtained in this study. Unlike previous studies that only reported the overall functional composition of fungal communities, the vertical distribution of functional guilds within the grain bulk was analyzed in this work. The results show that the functional potential of fungal communities varies significantly with grain bulk depth, with the upper layer having a higher potential for plant pathogenicity and the lower layer having a higher potential for saprotrophic spoilage. This functional differentiation provides a mechanistic explanation for the different types of quality deterioration that occur in different grain bulk depths. This spatial functional heterogeneity was not considered in most previous studies, by which an incomplete understanding of the spoilage mechanisms in stored grain may have been caused.

### 3.7. Screening of Dominant Spoilage Fungi in Stored Wheat

Based on the results of fungal diversity, community variation with grain bulk depth, functional prediction, and culturable fungi isolation, four core criteria were established to screen dominant spoilage fungi in stored wheat: (1) stable occurrence: strains belonging to *Ascomycota*, the absolutely dominant phylum accounting for 81.28% of the total fungal community, that were stably detected in all wheat samples; (2) ecological hazard: strains enriched in high-risk areas prone to wheat storage deterioration, or core stable taxa across different grain bulk depths; (3) deterioration potential: strains assigned to spoilage-related functional guilds (Pathotroph, Saprotroph or mixotroph) via FUNGuild prediction, with potential to induce wheat quality deterioration; and (4) high culturability: strains with high isolation frequency and colony abundance in culturable assays [25].

Finally, four fungal strains (*Aspergillus niger*, *Penicillium citrinum*, *Fusarium lateritium*, and *Talaromyces islandicus*) that met all the screening criteria were selected as test strains for subsequent artificial back-inoculation assays.

This study established a four-dimensional screening system for dominant spoilage fungi in stored wheat, which makes up for the deficiency of previous studies that only took abundance as the screening standard. The four selected strains cover the core spoilage fungal genera in the whole wheat storage cycle and have clear spoilage potential, which provides a scientific target selection for the subsequent artificial inoculation test.

Two main screening methods have been used in previous studies on dominant spoilage fungi in stored wheat: one is based solely on the isolation frequency of culturable fungi [26], and the other is based solely on the relative abundance in high-throughput sequencing data [5]. Both methods have significant limitations: the former underestimates the importance of unculturable or low-culturability fungi, while the latter cannot distinguish between active and dead fungi. Unlike these single-dimensional screening methods, a comprehensive four-dimensional screening system that combines both culture-dependent and culture-independent data, as well as functional information, was established in this work. This system makes up for the deficiency of previous studies that only took abundance as the screening standard. For example, *T*. *islandicus* has a relatively low relative abundance in sequencing data, but it was selected because it was stably detected in all samples, and mycotoxin production by this species has been reported. This more rigorous screening system ensures that the selected strains are truly representative of the dominant spoilage fungi in actual storage environments.

### 3.8. Changes in Physicochemical Properties of Wheat Infected by Dominant Fungi

**Figure 4:**
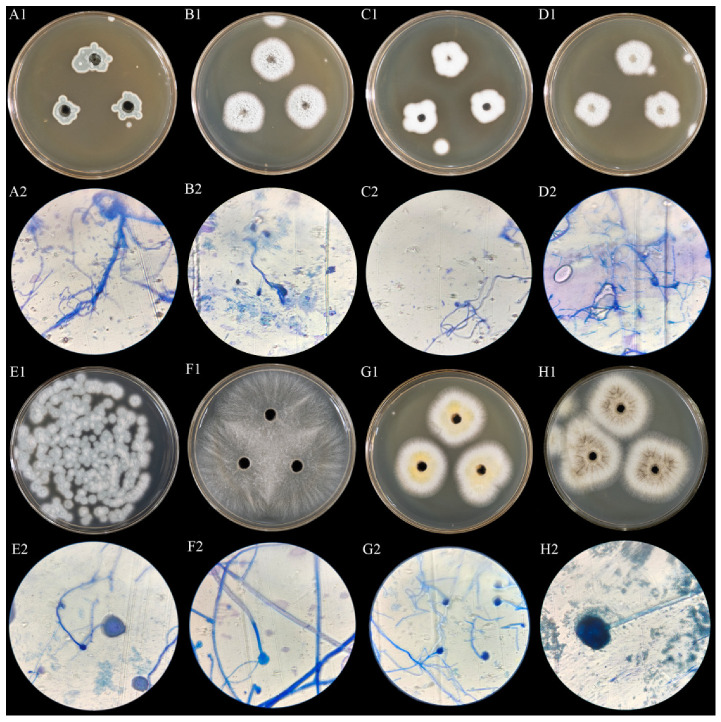
Colony characteristics and sporophore features of dominant fungi isolated and purified from wheat surface ((**A1**–**H1**) correspond to the colony characteristics of fungi A1–H1, respectively, and (**A2**–**H2**) correspond to the sporophore features of fungi A1–H1, respectively).

A total of eight culturable fungal strains were isolated and purified from wheat samples, numbered A1~H1. Their colony morphology and sporophore morphological characteristics under a 400× light microscope are shown in Figure 4. All strains were taxonomically identified via ITS rRNA gene sequencing and NCBI BLAST sequence alignment, with the detailed information shown in Table 1. The identification similarity of all eight strains was ≥99%. Combined with the pre-established screening criteria, four strains (*A. niger*, *F. lateritium*, *P. citrinum* and *T. islandicus*) with the highest isolation frequency and culturability were selected as test strains for subsequent artificial back-inoculation assays [26].

**Figure 5:**
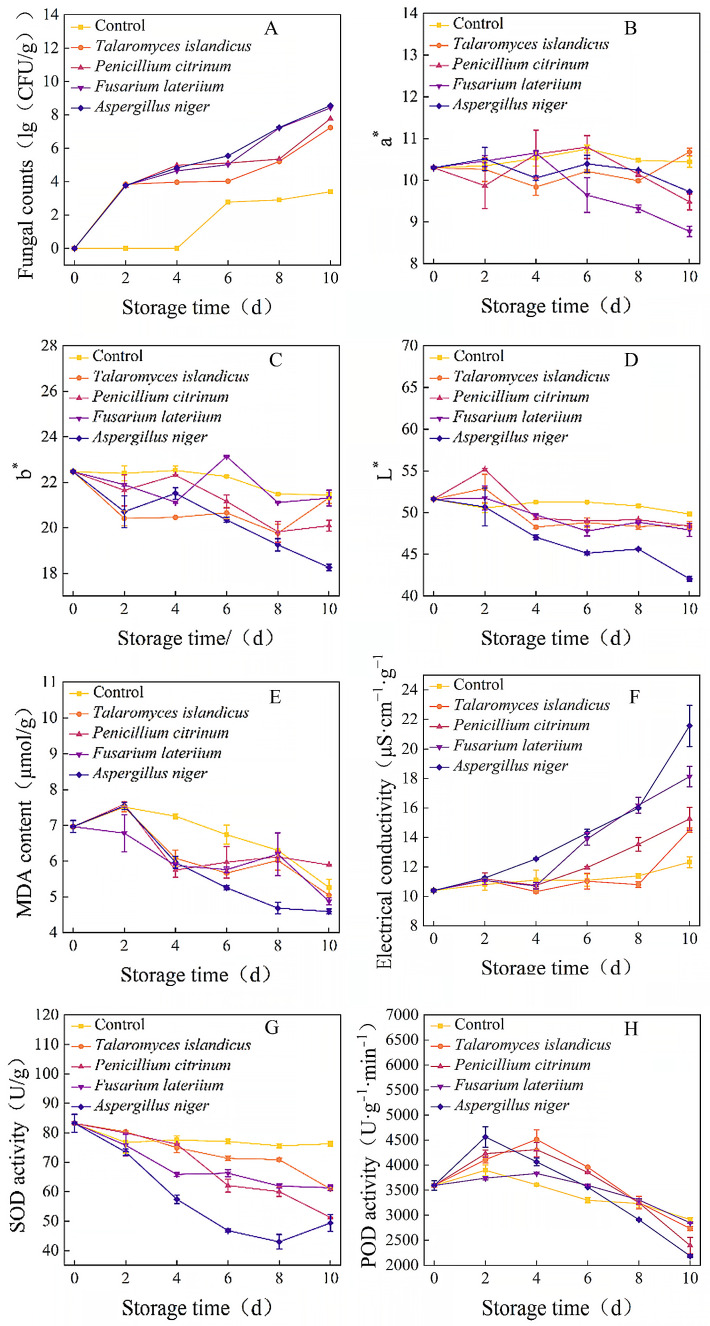
Changes in the total fungal colony count (**A**), a* value (**B**), b* value (**C**), L* value (**D**), malondialdehyde (MDA) content (**E**), electrical conductivity (**F**), superoxide dismutase (SOD) activity (**G**) and peroxidase (POD) activity (**H**) of wheat during storage after fungal inoculation.

All tested fungal strains proliferated rapidly after inoculation into wheat samples, but their growth rates on wheat kernels differed significantly (Figure 5A). *A. niger* and *F. lateritium* exhibited the fastest growth, with total fungal counts reaching 3.6 × 10^8^ CFU/g and 2.6 × 10^8^ CFU/g on day 10 of storage, respectively. This was followed by *P. citrinum* and *T. islandicus*, with total fungal counts of 6.0 × 10^7^ CFU/g and 1.7 × 10^7^ CFU/g after 10 days of storage, respectively.

No significant changes in L*, a* and b* values of wheat were observed in the non-inoculated control over the 10-day storage period (Figure 5B–D). The L* values of the *T. islandicus*, *P. citrinum* and *F. lateritium* groups increased slightly on day 2 of storage, followed by a continuous decreasing trend. *A. niger* caused the most severe reduction in wheat lightness, with its L* value decreasing from 51.62 to 42.06 during storage. The a* and b* values of all inoculated groups fluctuated with storage time, and the overall variation trend was consistent with the colony color of the corresponding inoculated strain [27,28].

Malondialdehyde (MDA) is a key product of cellular membrane lipid peroxidation, and its content can directly reflect the degree of plant cell membrane damage [29,30]. The MDA content in the control and all experimental groups showed a transient increase on day 2, followed by a gradual decreasing trend during storage (Figure 5E). Among all groups, *A. niger* had the greatest effect on wheat MDA content, which was significantly different from the control after 10 days of storage, with a decrease of 34% (*p* < 0.05). The electrical conductivity of wheat seed leachate after soaking can reflect the integrity of seed cell membrane to a certain extent [31,32]. The electrical conductivity of wheat samples increased with storage time in all inoculated groups (Figure 5F). Wheat inoculated with *A. niger* showed a significant difference from the control on day 4 (*p* < 0.05), and the *P. citrinum* and *F. lateritium* groups showed significant differences on day 6 (*p* < 0.05), whereas the *T. islandicus* group did not show a significant difference from the control until the 10th day of storage (*p* < 0.05). After 10 days of storage, the electrical conductivity of the *A. niger* group was significantly higher than that of all other groups (*p* < 0.05).

SOD is a key protective enzyme for plants to remove excess reactive oxygen species, which plays an important role in resisting external stress, reducing reactive oxygen species accumulation and maintaining cell membrane integrity [33,34]. Peroxidase (POD) is a key oxidoreductase in peroxisomes, which catalyzes substrate oxidation with hydrogen peroxide as the electron acceptor, and has the dual function of eliminating hydrogen peroxide and reducing the toxicity of harmful substances such as phenols and aldehydes [35,36,37]. In the non-inoculated control, the SOD activity of wheat samples remained stable during the 10-day storage period, with only a slight decrease from 83.19 U/g to 76.24 U/g, and no significant difference was observed during the whole storage period (Figure 5G). The SOD activity of the *T. islandicus*, *P. citrinum* and *F. lateritium* groups all decreased continuously during storage, with the lowest values of 60.93 U/g, 51.26 U/g and 61.29 U/g on day 10, respectively, which were significantly lower than those of the control (*p* < 0.05). The POD activity of wheat in all groups showed a trend of initial increase followed by a decrease, with the peak value appearing on days 2–4 of storage (Figure 5H). On day 10, the POD activity of all inoculated groups was significantly lower than that of the control (*p* < 0.05).

The artificial back-inoculation assay in this study confirmed that *A. niger* and *F. lateritium* exhibited the fastest growth and colonization rate on wheat kernels, which was highly consistent with the significant enrichment of *Aspergillus* and *Fusarium* in the high-mildew-risk lower grain bulk layer from our previous community survey, and aligned with Solanki et al.’s [4] conclusions on the wheat spoilage potential of these two genera. Unlike previous studies mostly focusing on the mycotoxin-producing properties of *Fusarium graminearum*, this study verifies the strong colonization ability of *F. lateritium* in stored wheat. Dynamic changes in SOD, POD, MDA and electrical conductivity essentially reflect the attack–defense interaction between dominant spoilage fungi and the wheat ROS metabolism system. In the early infection stage (0–2 d post-inoculation), fungal conserved pathogen-associated molecular patterns triggered wheat’s cascade antioxidant defense, explaining the transient POD activity peak and mild MDA rise [38]. During storage for 2–10 d, fast-growing *A. niger* and *F. lateritium* severely disrupted the wheat antioxidant system by secreting mycotoxins, cell-wall-degrading enzymes and virulence metabolites to inhibit SOD/POD activity [39,40], leading to excessive ROS accumulation and aggravated cell membrane damage, consistent with the continuous rise in electrical conductivity. Notably, the MDA content showed an initial increase followed by a decrease under fungal infection, revising the traditional cognition of a completely positive correlation between MDA and grain damage, and clarifying electrical conductivity as a more stable cell membrane damage indicator in fungal infection scenarios. *Penicillium citrinum* and *Talaromyces islandicus* showed weaker destructive capacity, confirming that fungal spoilage ability is directly correlated with their regulation of the SOD-POD-MDA system, which complements Attia et al.’s [41] multi-strain comparative study [42,43].

The finding that *A. niger* and *Fusarium* species have strong spoilage potential is consistent with the conclusions of Solanki et al. [4] on the wheat spoilage potential of these two genera. However, most previous studies on *Fusarium* in stored wheat have focused on *Fusarium graminearum* and its mycotoxin-producing properties [6,7]. Unlike these studies, the strong colonization ability of *F. lateritium* in stored wheat was verified, which is rarely reported in the previous literature. This finding expands the understanding of the spoilage potential of *Fusarium* species in stored grain. Regarding the physiological indicators of wheat damage, MDA was generally considered a reliable indicator of cell membrane damage in previous studies [29,30]. However, the results show that the MDA content decreases in the later stage of fungal infection, which revises this traditional cognition. It was clarified that electrical conductivity is a more stable cell membrane damage indicator in fungal infection scenarios, as it directly reflects the leakage of intracellular substances regardless of subsequent metabolic processes. This finding is of great importance for the establishment of accurate grain damage assessment methods. A multi-strain comparative study on the effects of fungi on plant antioxidant systems was conducted by Attia et al. [41], but the stability of different damage indicators was not compared. Their work is complemented by this study by identifying electrical conductivity as a superior indicator for fungal infection assessment.

### 3.9. Changes in Quality Properties of Wheat Infected by Dominant Fungi

**Figure 6:**
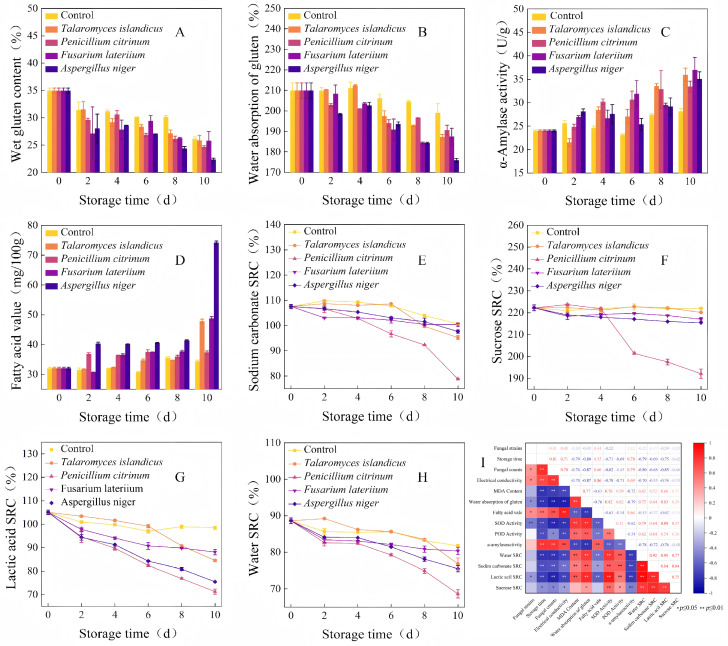
Changes in wet gluten content (**A**), water absorption of gluten (**B**), α-Amylase activity (**C**), fatty acid value (**D**), sodium carbonate SRC (**E**), sucrose SRC (**F**), lactic acid SRC (**G**) and water SRC (**H**). The correlation heatmap of physiological and storage quality indices of wheat during storage after fungal infection (**I**).

The wet gluten content and gluten water absorption of wheat decreased significantly in all groups during storage (Figure 6A,B, *p* < 0.05). On day 10, the wet gluten content was reduced by 8.82–12.63 percentage points across groups, with the largest decline observed in the *A. niger* group. Gluten water absorption dropped from the initial value of 209.84% to 175.91–198.99% in all groups; the *A. niger* group fell below the national storage suitability threshold (180%) on day 10 [44,45].

Wheat α-amylase activity showed an overall upward trend in all groups, with a 17% increase in the control over 10 days (Figure 6C, *p* < 0.05). *F. lateritium* and *T. islandicus* caused the greatest increase (54% and 50% increase, respectively), and all fungal treatments except *P. citrinum* significantly increased enzyme activity (*p* < 0.05).

Wheat fatty acid value increased continuously with the extension of storage time in all groups (Figure 6D, *p* < 0.05). In the non-inoculated control group, the fatty acid value rose slightly from 32.05 mg/100g to 35.62 mg/100g on day 8 of storage. In contrast, all fungal inoculation treatments induced a significant and continuous elevation in wheat fatty acid value throughout storage. *A. niger* caused the most dramatic increase, reaching 74.20 mg/100g on day 10 of storage (a 132% increase compared with the initial value), far exceeding the national standard threshold for storage suitability. *F. lateritium*, *P. citrinum* and *T. islandicus* caused significantly lower elevation in fatty acid value compared with *A. niger* (*p* < 0.05).

All four SRC indicators (water, sucrose, lactic acid, and sodium carbonate) of wheat showed an overall downward trend during storage, with a significantly smaller decline in the non-inoculated control (Figure 6E–H, *p* < 0.05). The *P. citrinum* group had the largest decline in all SRC indicators, with values significantly lower than other groups (*p* < 0.05).

Correlation heatmap analysis was performed to clarify the link between fungal growth and wheat quality deterioration (Figure 6I). The total fungal colony count was significantly positively correlated with electrical conductivity, fatty acid value and α-amylase activity (*p* < 0.01) and significantly negatively correlated with gluten water absorption, antioxidant enzyme activity and all SRC indicators. Gluten water absorption had the strongest correlation with total fungal count (r = −0.87, *p* < 0.01).

The differential damage characteristics of four dominant spoilage fungi on the storage and processing quality of wheat were clarified in this study. The strongest destructive effect on core wheat storage quality indicators, including gluten water absorption and fatty acid value, was exerted by *A. niger*. Dramatic fatty acid elevation was driven by *A. niger* via secretion of high-activity extracellular lipase, destroying oil body integrity and aggravating lipid oxidative rancidity [46]. Meanwhile, the most significant effect on four wheat processing quality SRC indicators was exhibited by *P. citrinum* despite weaker growth. This indicates that different fungal species have different target preferences for wheat quality damage: *A. niger* mainly affects storage quality, while *P. citrinum* mainly affects processing quality. It was confirmed by correlation analysis that gluten water absorption had the strongest correlation with total fungal load (r = −0.87), which is higher than the correlation coefficient of the traditionally used fatty acid value (r = 0.79). This indicates that gluten water absorption is a more sensitive early warning indicator for fungal infection in wheat than fatty acid value. This result provides critical theoretical support for the targeted fungal control of stored wheat.

The finding that *A. niger* is the core genus driving lipid deterioration in stored wheat is consistent with the conclusion of Alcon et al. [47]. However, only the qualitative effect of *Aspergillus* on fatty acid value was reported in previous studies, without quantifying the change threshold under single-strain infection. Unlike these studies, it was further quantified that *A. niger* can increase the fatty acid value of wheat to 74.20 mg/100g within 10 days under the experimental conditions, which is 2.3 times the initial value and far exceeds the national storage suitability threshold. This quantitative data provides a clear reference for grain storage quality assessment. Regarding the effect of fungi on wheat processing quality, previous studies have mainly focused on the wet gluten content and dough rheological properties [48,49,50]. It was found in this study that *P. citrinum* exerted the most significant effect on the four SRC indicators of wheat flour, which is a novel finding. SRC indicators reflect the functional properties of wheat flour components (starch, protein, and pentosan), and their changes directly affect the processing quality of wheat products such as bread and noodles. This finding highlights the importance of controlling *Penicillium* contamination to maintain wheat processing quality [49]. Most importantly, gluten water absorption was identified as a more sensitive early warning indicator for wheat fungal infection than the traditionally used fatty acid value. This finding challenges the current grain storage quality assessment system, which mainly relies on fatty acid value. Earlier detection of fungal contamination can be enabled by the higher sensitivity of gluten water absorption, allowing for timely intervention to prevent further quality deterioration.

## 4. Conclusions

In this study, high-throughput sequencing was used to characterize the fungal community structure on the surface of stored wheat kernels and across different grain bulk depths, and the vertical distribution pattern of fungal communities in wheat bulk was clarified. *Ascomycota* and *Basidiomycota* were the dominant fungal phyla on wheat surfaces, and *Aspergillus* was significantly enriched in the lower grain layer with high risk of fungal spoilage, with a relative abundance of up to 19.44%. Furthermore, we established a targeted, quantifiable screening system for core dominant spoilage fungi in stored wheat and screened four core spoilage strains: *Aspergillus niger*, *Fusarium lateritium*, *Penicillium citrinum* and *Talaromyces islandicus*. Back-inoculation assays confirmed that all four strains caused wheat physiological and quality deterioration with significant interspecific differences: *A. niger* exerted the most severe destructive effect on wheat storage quality, *F. lateritium* severely impaired the integrity of wheat cell membrane and antioxidant defense system, and *P. citrinum* mainly affected the processing quality of wheat. Notably, gluten water absorption was identified as a sensitive early warning indicator for wheat fungal infection, which showed the strongest correlation with total fungal count (r = −0.87).

Future research will focus on verifying the spoilage effect of dominant fungi under actual grain depot storage conditions and on exploring the synergistic spoilage mechanism of multiple fungal strains in complex storage environments. Meanwhile, we will develop a rapid on-site detection method based on the gluten water absorption early warning indicator, and carry out research on targeted green biocontrol technologies for core spoilage fungi, to provide comprehensive technical support for early warning of fungal spoilage and quality safety assurance during wheat storage.

## Figures and Tables

**Table 1:** Morphological characteristics and identification results of fungi isolated from wheat samples.

Serial Number	GenBank Accession No.	Colony Morphology	Microscopic Characteristics	BLAST Comparison Results	Similarity
A	ON28466.1	Yellow-orange colony with floccose to felt-like texture, neat margins, slow growth.	Short conidiophores; elliptical conidia arranged in chains.	*Talaromyces islandicus*	≥99%
B	MW113441.1	Initially white, later turning orange-yellow, floccose, with radial wrinkles at the margin; yellow reverse.	Conidiophores with penicillus-like branching; spherical to sub-spherical conidia.	*Penicillium citrinum*	≥99%
C	MH865265.1	White to cream-colored, floccose, neat margins, slightly yellowish in later stages, fast growth.	Radiate conidial heads; spherical vesicles; elliptical, smooth-surfaced conidia.	*Aspergillus candidus*	≥99%
D	OP4283.1	Brownish-yellow to brown, felt-like texture, irregular margins, wrinkled surface.	Long and branched conidiophores; slender elliptical conidial chains.	*Talaromyces pseudofuniculosus*	≥99%
E	MN958046.1	Blue-green to dark blue, floccose, with a surrounding white mycelial ring at the margin; yellow-brown reverse.	Columnar conidial heads; flask-shaped vesicles; spherical, rough-surfaced conidia.	*Aspergillus welwitschiae*	≥99%
F	OM900821.1	Pink to brick-red, floccose, neat margins, deep brick-red reverse.	Sickle-shaped multiseptate macroconidia; elliptical clustered microconidia.	*Fusarium lateritium*	≥99%
G	KM203615.1	Yellow to brown, felt-like texture, irregular margins, raised surface.	Short conidiophores; short and dense conidial chains; round conidia.	*Talaromyces radicus*	≥99%
H	ON208675.1	Black to dark brown, floccose, with mixed white mycelium at the margin; pale yellow reverse.	Spherical conidial heads; large vesicles; spherical, rough-surfaced conidia in chains.	*Aspergillus niger*	≥99%

## Data Availability

The original contributions presented in the study are included in the article; further inquiries can be directed to the corresponding authors.
